# What shapes mathematics anxiety in K-12 education? A scoping review of research from 2019 to 2025

**DOI:** 10.3389/fpsyg.2026.1894090

**Published:** 2026-08-06

**Authors:** Yu Zhou, Bin Jing, Qin Wang, Jing Zhang

**Affiliations:** 1 Faculty of Education, Shaanxi Normal University, Xi’an, Shaanxi, China; 2 Xinjiang Basic Education Quality Improvement Research Center, Changji University, Changji, Xinjiang, China; 3 School of Mathematics and Data Science, Changji University, Changji, Xinjiang, China

**Keywords:** influencing factors, interventions, K-12 education, mathematics anxiety, methodological characteristics

## Abstract

**Introduction:**

Despite growing interest in mathematics anxiety, comprehensive syntheses of recent K–12 research remain limited. This scoping review aims to map the literature on mathematics anxiety that was published between January 2019 and December 2025.

**Methods:**

Following PRISMA-ScR guidelines, this review analyzed 132 empirical studies. The analysis categorized influencing factors into antecedents, processes, and outcomes, while also examining methodological characteristics and interventions.

**Results:**

Findings indicate that antecedent factors comprise individual (e.g., gender, grade) and environmental influences (e.g., parents, teachers, peers, culture), though research on high school students and cross-national comparisons remains notably underdeveloped. Process factors encompass behavioral, cognitive, and emotional dimensions, with cognitive aspects dominating the literature. Mathematics performance persists as the primary outcome of concern. Methodologically, studies rely heavily on variable-centered, cross-sectional designs, with limited use of person-centered, longitudinal, or multimodal approaches. Interventions predominantly target either contextual restructuring (e.g., peer tutoring, game design, mathematical software) or strategy training (e.g., skill enhancement, emotional regulation).

**Discussion:**

These findings highlight the need for integrated systemic models and methodological pluralism. Effective interventions must pair anxiety-reducing classrooms with self-regulation training, with particular urgency for high school students facing critical transitions.

## 1 Introduction

The Organization for Economic Cooperation and Development reports that over 33% of young learners experience mathematics anxiety, characterized by fear, tension, avoidance, and feelings of inferiority toward mathematics (Ahmed, 2018; Namkung et al., 2019; PISA, 2016). Empirical studies have also documented mathematics anxiety as a salient affective factor co-occurring with motivational and cognitive variables (Abín et al., 2020). This emotion negatively affects short-term mathematics learning and may have long-term implications for academic achievement and career development (Eidlin-Levy et al., 2023; Mammarella et al., 2023). Consequently, understanding and mitigating mathematics anxiety are key research priorities in educational psychology and mathematics education.

Researchers have conducted reviews examining mathematics anxiety’s causes, development, and intervention approaches (Codding et al., 2023; Luttenberger et al., 2018; Shore and Kelleher, 2024). Some reviews summarized the definition, measurement, influencing factors, intervention measures, and explanatory framework of mathematics anxiety (Dowker et al., 2016; Luttenberger et al., 2018; Ramirez et al., 2018). Some reviews focused on specific topics (Barroso et al., 2021; Balt et al., 2022; Codding et al., 2023; Shore and Kelleher, 2024), such as the predictive role of early mathematics anxiety intervention measures, and the relationship between mathematics anxiety and mathematics academics and their moderating factors (Barroso et al., 2021; Balt et al., 2022; Caviola et al., 2022). Notably, the direction of causality between mathematics anxiety and performance remains contested. Carey et al. (2016) synthesized evidence for two predominant accounts: the deficit theory, in which low mathematical competence fosters anxiety, and the debilitating anxiety model, in which anxiety impairs performance. Crucially, these pathways are not mutually exclusive but may operate reciprocally over time. However, most extant research adopts a unidimensional analytical perspective and does not fully conceptualize mathematics anxiety as an endogenous affective variable within a multidimensional “antecedent–process–outcome” system. Moreover, methodological limitations have constrained understanding of the mechanisms through which mathematics anxiety operates. At the same time, ambiguity surrounding intervention mechanisms continues to hinder treatment innovation. Therefore, examining the correlates and methodological characteristics of mathematics anxiety, and clarifying its intervention mechanisms within an integrative, multidimensional framework, is essential for advancing both theory and practice in this field (Cevikbas et al., 2024).

To achieve the above goals, this study employs a scoping review method to systematically analyze empirical research on K-12 mathematics anxiety from January 2019 to December 2025. Following scoping review protocols, we (1) define research questions, search strategies, and eligibility criteria; (2) extract and synthesize literature; and (3) analyze influencing factors, methodologies, and interventions using the CILS 2018 framework (Fraillon et al., 2018). This approach identifies research focus and gaps while offering systematic guidance for future work.

## 2 Literature review

### 2.1 The relationship between mathematics anxiety and other concepts

Mathematics anxiety refers to the tension and unease experienced during mathematical activities in academic and everyday contexts (Ashcraft, 2002; Richardson and Suinn, 1972). Specifically, students with mathematics anxiety often avoid mathematics learning, engage in less deep thinking and practice, weakening their mathematical skills and performance (Codding et al., 2023; Mammarella et al., 2023). This anxiety may also lead to avoidance of careers that require advanced mathematical skills, limiting career choices and professional growth opportunities (Ahmed, 2018; Eidlin-Levy et al., 2023).

Previous research has consistently identified key factors influencing mathematics anxiety. At the individual level, gender and grade level were significant predictors, with girls typically exhibiting higher mathematics anxiety than boys (Xie et al., 2019; Van Mier et al., 2019), and anxiety increasing with grade level (Ahmed et al., 2012; Balt et al., 2022). Environmental factors, such as family, school, and cultural contexts, also played crucial roles (Jia and Cheng, 2024; Wang et al., 2021; Wang et al., 2025). Parental mathematics anxiety might transfer to children, while teacher and peer support helped reduce math-related stress (He et al., 2025; Szczygieł, 2020; Kim et al., 2023). Cross-cultural studies revealed that the mathematics anxiety-performance relationship was particularly strong in Asian countries like China (Ching, 2017; Zhang et al., 2019).

In addition, mathematics anxiety also significantly affected students’ mathematics performance, skill acquisition, ability development, cognitive processing, problem-solving strategies, and career orientation (Barroso et al., 2021; Eidlin-Levy et al., 2023; Zhu et al., 2024). Extensive research demonstrated that mathematics anxiety was significantly negatively correlated with academic performance (Barroso et al., 2021; Namkung et al., 2019), and significantly reduced students’ mathematics engagement while adversely affecting their learning motivation (Quintero et al., 2022). In fact, social cognitive theory and control value theory of emotions were often used to analyze its causes and mechanisms of action in exploring the relationship between mathematics anxiety and multiple factors (Dowker et al., 2016; Wang et al., 2021; Zhou et al., 2025). Social cognitive theory emphasizes the interaction between individual cognition, behavior, and environment (Bandura, 2002). For example, parents’ mathematics anxiety may be transmitted to children through observational learning mechanisms. The control-value theory of emotions proposes that anxiety arises from perceptions of control and task value, subsequently influencing motivation (Pekrun, 2006). For example, students with mathematics anxiety may reduce their learning investment due to excessive focus on potential failures, initiating a cognitive-emotional-behavioral cycle that undermines academic growth. Supporting this developmental interplay (Barroso et al., 2021). Song et al. (2021) demonstrated in a longitudinal study that young students’ feelings about mathematics and their arithmetic performance reciprocally predict one another over time. Collectively, these findings suggest that mathematics anxiety emerges from a complex interplay between external environmental factors and internal cognitive-behavioral processes. Thus, analyzing antecedents, emotional development processes, and their behavioral and academic outcomes can reveal the mechanisms underlying mathematics anxiety.

### 2.2 The research methodology of mathematics anxiety

There were several forms of mathematics anxiety analysis methods (Williams et al., 2024). The variable-centered method assumes consistent relationships between mathematics anxiety and other variables (e.g., performance, self-efficacy) across individuals (Cribbs et al., 2021; Weissgerber et al., 2022). Furthermore, some studies further assume students with different levels of mathematics anxiety groups, potentially yielding different relationships with other variables (Ashkenazi and Cohen, 2023; González-Gómez et al., 2023). For instance, Ashkenazi and Cohen (2023) found that students with high mathematics anxiety showed less developed numerical estimation skills. In addition, the person-centered approach assumes individual differences in these relationships (Hickendorff et al., 2018; Williams et al., 2024). For instance, Broda et al. (2023) applied latent profile analysis to identify five distinct ninth-grade profiles combining mathematics anxiety, self-concept, and interest levels, revealing that high anxiety doesn’t necessarily correlate with low self-concept or interest.

There were many different research methods for investigating mathematics anxiety. Most quantitative research used questionnaires to analyze its influencing factors (Jason and Glenwick, 2016). For example, Zhou et al. (2025) employed questionnaires to assess teacher support and mathematics anxiety, revealing environmental factors’ influence on anxiety levels. Qualitative research typically explored individual experiences, causes, and mechanisms through interviews (Hammarberg et al., 2016; Jason and Glenwick, 2016). For example, Petronzi et al. (2024) interviewed students about effective learning methods and found that storybooks alleviate mathematics anxiety. Mixed-method research combines the advantages of the two methods to improve the depth and reliability of the results (Creswell, 2009; Jason and Glenwick, 2016). For example, Demirtaş and Uygun-Eryurt (2022) found that hope and school climate mediate the parental attachment mathematics anxiety relationship, with qualitative interviews supporting these quantitative findings. From a temporal perspective, cross-sectional studies collect data at specific time points to reveal interrelationships (Spector, 2019). For example, Broda et al. (2023) pointed out that positive math self-concept is associated with low anxiety through a single survey data. Longitudinal studies track long-term data to explore causal relationships (Zapf et al., 1996). For example, Sorvo et al. (2022) found that mathematics anxiety changes with changes in math self-concept and values. From the perspective of research design and data collection, survey studies explore subjective experiences through questionnaires (Namkung et al., 2019) and reveal their relationship with academic performance, gender and other factors (Barroso et al., 2021; Namkung et al., 2023; Wang et al., 2021). Experimental studies evaluate the effectiveness of interventions and provide a basis for formulating strategies (Moliner and Alegre, 2020; Passolunghi et al., 2020). For example, Moliner and Alegre (2020) showed that peer tutoring can reduce mathematics anxiety among middle school students.

Mathematics anxiety was primarily measured using standardized scales, with the Mathematics Anxiety Rating Scale (MARS) being the most established (Richardson and Suinn, 1972). Commonly used variants include the 25-item sMARS (Alexander and Martray, 1989) and 9-item AMAS (Hopko et al., 2003), both internationally validated for different age groups. The PISA project’s concise four-item scale is particularly effective for large-scale studies (PISA, 2016). However, self-report scales may be limited by subjective bias (Winkielman et al., 2011). To address this, physiological measures like heart rate monitoring, EEG, and fMRI (Klados et al., 2019; Pizzie et al., 2020) objectively quantify anxiety responses to mathematics stimuli, revealing its physiological and cognitive mechanisms (Kreibig, 2010).

### 2.3 The treatment of mathematics anxiety

Extensive research has demonstrated that mathematics anxiety negatively impacts students’ immediate and long-term mathematical development (Sorvo et al., 2022; Namkung et al., 2019). Notably, Koponen et al. (2024) emphasized that such effects are particularly evident in individual responses to arithmetic fluency interventions. Therefore, mastering effective mitigation strategies is essential. Proven strategies included expressive writing, reappraisal, relaxation techniques, cooperative learning, targeted tutoring, and digital interventions focusing on number sense and executive functions (Codding et al., 2023; Passolunghi et al., 2020). Existing interventions have been typically categorized into two categories: one is a mathematics ability intervention based on the “*reduced competency account*” interpretation, and the other is a cognitive behavioral intervention based on the “*disruption account* “interpretation (Dowker et al., 2016; Balt et al., 2022). In addition, recent studies by Balt et al. (2022) summarized interventions for school-age children, and Codding et al. (2023) found through meta-analysis that therapeutic interventions were more effective in reducing mathematics anxiety symptoms than simple mathematics skill interventions. These studies provide valuable insights into finding effective interventions to reduce children’s mathematics anxiety.

### 2.4 The review gap of mathematics anxiety in K-12 mathematics education

Literature reviews on mathematics anxiety have systematically advanced this research field. Dowker et al. (2016) synthesized six decades of research to define mathematics anxiety, differentiate it from other anxieties, examine its relationship with mathematical attitudes, and introduce measurement approaches. Their analysis also identified key influencing factors (genetics, gender, age) and effective interventions (mathematics tutoring, expressive writing). Luttenberger et al. (2018) expanded this foundation by analyzing environmental and individual influences, demonstrating long-term effects, and emphasizing measurement’s critical role in mitigation. Ramirez et al. (2018) introduced a novel framework highlighting the dual importance of understanding and addressing mathematics anxiety. Recent review researches have addressed specialized aspects of mathematics anxiety. For instance, Zhang et al. (2019) and Barroso et al. (2021) quantified its negative effects on academic achievement and identified moderators including geographical regions, grade level, and measurement. O’Hara et al. (2022) examined how classroom environments affect mathematics anxiety, whereas Codding et al. (2023) assessed school interventions for reducing it. Dondio et al. (2023) examined game-based approaches, and Shore and Kelleher (2024) established the efficacy of early interventions for elementary students. Together, these studies have substantially advanced the field’s understanding of mathematics anxiety.

**FIGURE 1:**
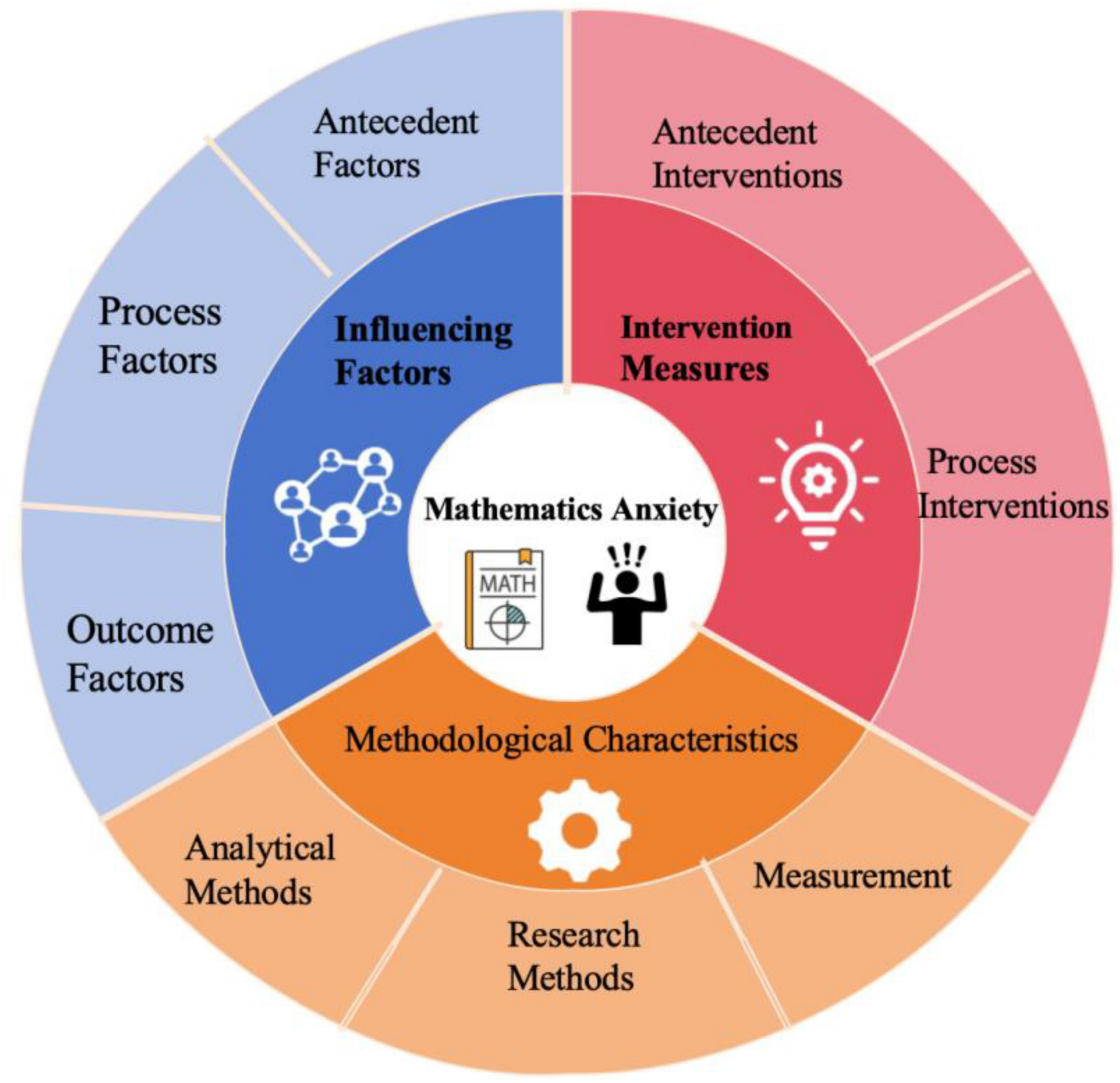
The analytical framework of the present study.

Most existing review studies summarized and discussed the influencing factors of mathematics anxiety through a unidimensional factor perspective (Dowker et al., 2016; Luttenberger et al., 2018; Ramirez et al., 2018). However, drawing on the ICILS 2018 framework and prior research, this endogenous emotional variable should be analyzed through an “antecedent-process-outcome” system that involves multidimensional factor interactions (Fraillon et al., 2018; Luttenberger et al., 2018; Ramirez et al., 2018). Antecedent factors represent contextual variables independent of learning processes or outcomes. Process factors, shaped by antecedents, directly affect math anxiety, while outcome factors are both influenced by and reciprocally interact with process factors (Fraillon et al., 2018; Luttenberger et al., 2018). This system shows antecedents shaping mathematics anxiety development, processes responding to existing anxiety levels, and outcomes continuously interacting with these processes. Moreover, current reviews suffer from methodological limitations, and expanding research approaches would enable deeper analysis of mathematics anxiety. While existing interventions are understood, updating these approaches and clarifying their mechanisms can help people design intervention measures (Codding et al., 2023; Shore and Kelleher, 2024). Therefore, reviewing the research on K-12 mathematics anxiety can help advance its theoretical and practical development (Cevikbas et al., 2024). Building on the ICILS 2018 framework and integrating previous ideas, the analytical framework in Figure 1 is proposed to better understand mathematics anxiety in K-12 education from 2019 to 2025.

### 2.5 The present study

Based on the analysis and synthesis of key empirical studies (January 2019 to March 2025), this study aims to address three core issues about mathematics anxiety in K-12 education:

(1) What are the influencing factors of mathematics anxiety research (antecedent factors, process factors, outcome factors)?
(2) What are the methodological characteristics of mathematics anxiety research (analysis methods, research methods, and measurements)?
(3) What are the intervention measures for mathematics anxiety research (antecedent interventions, process interventions)?

## 3 Methods

We constructed our scoping review study following the guidelines of Munn et al. (2018) and adhering to the Preferred Reporting Items for Systematic Reviews and Meta-Analyses Extension for Scoping Reviews (PRISMA-ScR; Peters et al., 2020). A scoping review serves as a precursor to systematic reviews or meta-analyses by identifying the scope of a literature body and providing an overview (Munn et al., 2018). Its purpose is to identify available evidence types, clarify key concepts and dimensions, examine research methodologies, highlight key characteristics or factors related to a concept, and analyze knowledge gaps.

### 3.1 Search procedure and eligibility criteria

**FIGURE 2:**
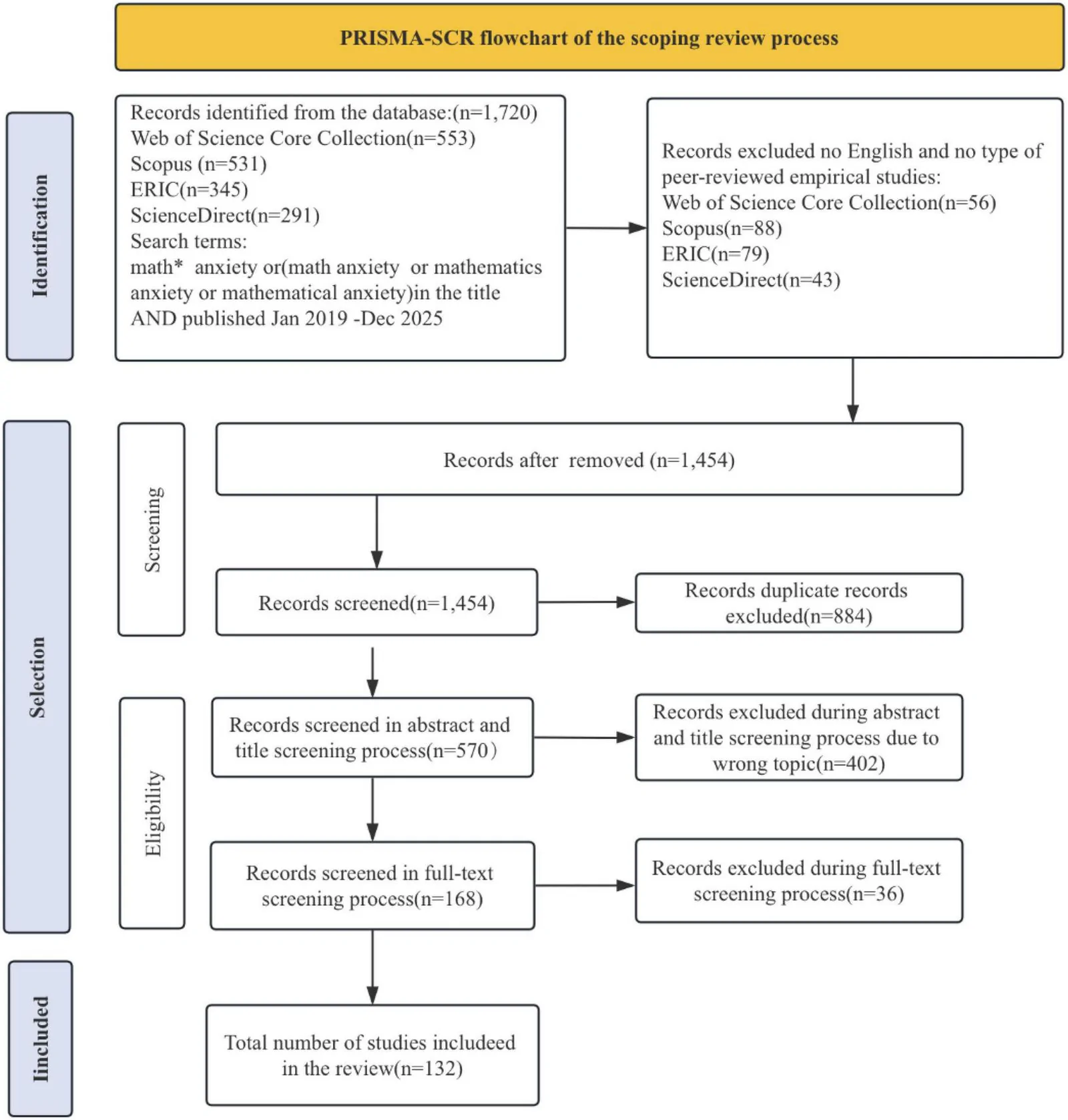
The PRISMA-SCR flowchart of the article selection process.

Web of Science (WoS) is a leading source of high-quality reviews and bibliometric data (Korom, 2019). It facilitates efficient literature searches, supports various informational needs, and aids in research topic mapping, trend monitoring, and academic activity analysis (Birkle et al., 2020). This review conducted an extensive literature search within the WoS electronic database. Using the Web of Science Core Collection, we searched for articles with the terms “math* anxiety,” “mathematics anxiety,” or “mathematical anxiety” in their titles between January 2019 and December 2025. To broaden our coverage, we also searched Scopus, ERIC, and ScienceDirect using the same terms and timeframe. Across these four databases, we identified a total of 1,720 articles. The research team followed a four-stage process to identify suitable records for this study (see Figure 2) to eliminate duplicates and articles that did not meet the publication type criteria (Table 1). Initially, the search was restricted to peer-reviewed empirical reports published in English, resulting in 1,454 articles. Then, manual screening and deduplication reduced this number to 570 articles for further analysis. After reviewing titles and abstracts, 168 articles were retained. Finally, a full-text review identified and coded 132 articles.

**TABLE 1:** Inclusion and exclusion criteria.

Inclusion criteria	Exclusion criteria
1 The studies focused on mathematics anxiety	The studies were not related to mathematics anxiety (e.g., test anxiety, classroom anxiety, academic anxiety, anxiety with other subjects)
2 The studies meet the definition of mathematics anxiety for students	The studies do not meet the definition of mathematics anxiety for students (e.g., studies on parental educational anxiety, parental mathematics anxiety, teacher mathematics anxiety)
3 The studies include samples in K-12	Studies excluding other samples (e.g., early childhood education, graduate student education, higher education teacher education, teachers, adults, parents)
4 Empirical peer-reviewed studies	Theoretical papers, editorials, books, book reviews, commentaries, notes, meeting abstracts, literature reviews, meta-analyses, review papers
5 The studies published in English	The studies published in a language other than English
6 The studies indexed in web of science core collection, scopus, ERIC, and ScienceDirect	The studies indexed in databases other than the web of science core collection, scopus, ERIC, and ScienceDirect
7 The Studies published between January 2018 and March 2025	The studies published before 2019

### 3.2 Coding process and data analysis

To assess mathematics anxiety among K-12 students comprehensively, we developed a coding manual based on the ICILS 2018 framework and a coding strategy based on previous review ideas (see Supplementary material). The process was as follows. Four researchers initially created a draft code by reviewing all articles, then collectively established coding categories and themes through discussion. Both researchers underwent 2 h of training, mastering the coding manual through practice simulations, and began formal coding only after achieving >95% accuracy. To ensure coding reliability, two researchers independently coded the same 26 randomly selected reports (approximately 20%), and interrater reliability was assessed using Cohen’s Kappa, which indicated high agreement (Cohen’s Kappa = 0.867, *p* < 0.001). Any inconsistencies were resolved through discussion, and after finalizing the coding scheme, one coder was responsible for coding the remaining articles.

## 4 Results

### 4.1 The study characteristics

Analysis of 132 studies revealed key trends in mathematics anxiety research (Table 2). Between 2019 and 2025, the volume of published papers on this topic surged, reflecting widespread concern about mathematics anxiety among students. Geographically, research concentrated on China, the United States, and Italy, with only three cross-national studies. While studies spanned K-12 populations, high school students (12.88%) and cross-grade comparisons (12.12%) were underrepresented. Sample sizes varied, with 28.78% including 201–500 participants, 24.24% exceeding 1,000, and 3.79% below 50. Among them, the phenomenon of fewer large sample, cross-national, high school, and cross-grade studies deserves further attention.

**TABLE 2:** Summary of the identified studies.

Category	Code	Frequency count	Percentage
Publication date	2019	9	6.82%
	2020	21	15.91%
	2021	14	10.61%
	2022	22	16.67%
	2023	23	17.42%
	2024	32	24.24%
	2025	11	8.33%
Country/region	China	29	21.97%
	US	20	15.15%
	Italy	10	7.58%
	Spain	7	5.30%
	Germany	5	3.79%
	Turkey	5	3.79%
	Australia	4	3.03%
	Indonesia	3	2.27%
	Countries	3	2.27%
	Others countries	45	34.09%
Participants	Elementary school students	65	49.24%
	Middle school students	34	25.76%
	High school students	17	12.88%
	Mix students	16	12.12%
Sample	0–50	5	3.79%
	51–100	9	6.82%
	101–200	29	21.97%
	201–500	38	28.79%
	501–1,000	12	9.09%
	> 1,000	32	24.24%

These categories are only coded if relevant.

### 4.2 The influencing factors of mathematics anxiety research

**FIGURE 3:**
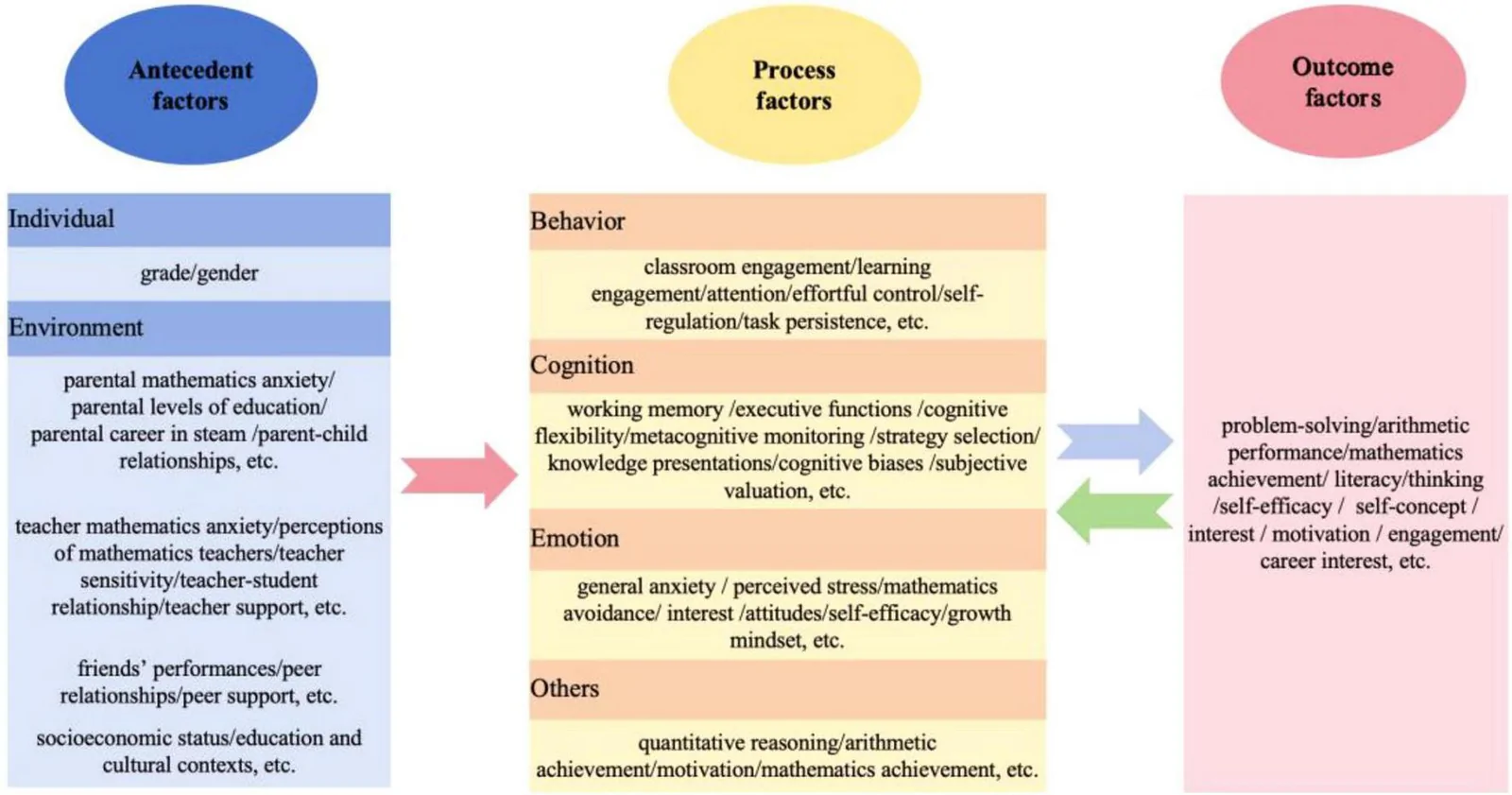
The influencing factors for mathematics anxiety. The double arrows between process factors and outcome factors emphasize the possibility of mutual correlation between process factors and outcome factors. The single arrow between antecedent factors and process factors indicates that the ICILS framework assumes a unidirectional influence between these two types of factors.

We analyzed 132 studies on mathematics anxiety in K–12 education. Grounded in the ICILS 2018 framework and prior research, we categorized the influencing factors into three groups (see Figure 3): antecedent, process, and outcome factors (Fraillon et al., 2018; Luttenberger et al., 2018). Antecedent factors were external background influences that shape mathematics anxiety development, unaffected by learning processes or outcomes. These included individual factors (e.g., grade, gender) and environmental factors (e.g., parental factors in the family, teacher factors in school, peer factors, and social factors). Process factors directly influenced mathematics anxiety while being shaped by antecedent factors. Using an analytical framework based on control value theory (Pekrun, 2006), these variables were systematically classified and coded into three categories: behavior, cognition, emotion, and others. Specifically, Behavioral factors encompass learning engagement and persistence (e.g., classroom engagement, task persistence). Cognitive factors included mathematical intelligence, reasoning skills, and self-assessment abilities (e.g., working memory, metacognition monitoring, subjective valuation). Emotional factors involved math-related attitudes and feelings (e.g., general anxiety, mathematics interest, mathematics avoidance). Additional factors included skills, knowledge, learning opportunities, time management, task difficulty, and resilience. Outcome factors represented the direct consequences of mathematics anxiety, including mathematics ability, learning interest, and career (e.g., mathematics problem-solving, achievement, career interest). Analyzing these factors reveals key insights into how mathematics anxiety develops.

### 4.3 The methodological characteristics of mathematics anxiety

Analysis of research methods in mathematics anxiety studies reveals several key patterns (Table 3). Most studies (78.03%) used variable-centered analyses, with a small portion (5.30%) applying person-centered latent profile analysis to examine group differences in anxiety levels and their relationship to other factors. Quantitative approaches dominated (97.73%), primarily using questionnaires, while only 2.27% employed mixed methods. The cross-sectional designs were most common (75.76%), compared to 24.24% of longitudinal studies tracking anxiety changes over time. Data collection relied heavily on surveys (81.06%), with experiments accounting for 18.94%. Self-report measures predominated, primarily using: Abbreviated Mathematics Anxiety Scale (28.79%) and PISA background questionnaire (13.64%). Notably, five studies incorporated physiological measurements for objective data.

**TABLE 3:** Distribution of methodological characteristics of identified studies.

Category	Code	Count	Percentage
Analysis methods	Variable-centered no classification	103	78.03%
	Variable-centered classification	21	15.91%
	Person-centered classification	7	5.30%
Research methods: 1	Quantitative	129	97.73%
	Qualitative	0	0.00%
	Mixed	3	2.27%
Research methods: 2	Cross-sectional studies	100	75.76%
	Longitudinal studies	32	24.24%
Research methods: 3	Experiment	25	18.94%
	Investigation	107	81.06%
Scale of mathematics anxiety	AMAS (Hopko et al., 2003)	38	28.79%
	PISA	18	13.64%
	MARS-E (Suinn et al., 1988)	8	6.06%
	MASC (Chiu and Henry, 1990)	8	6.06%
	MARS (Richardson and Suinn, 1972)	6	4.55%
	SEMA (Wu et al., 2012)	5	3.79%
	AEQ (Pekrun et al., 2011)	5	3.79%
	CMAQ-R (Ramirez et al., 2016)	4	3.03%
	MARS-R (Plake and Parker, 1982)	4	3.03%
	MAQ (Thomas and Dowker, 2000)	3	2.27%
	Others	37	28.03%
Physiological measures	EEG/FMRI/EDA/GSR/ ERE-TRACKING	5	3.79%

These categories are only coded if relevant.

### 4.4 The interventions in mathematics anxiety research

**FIGURE 4:**
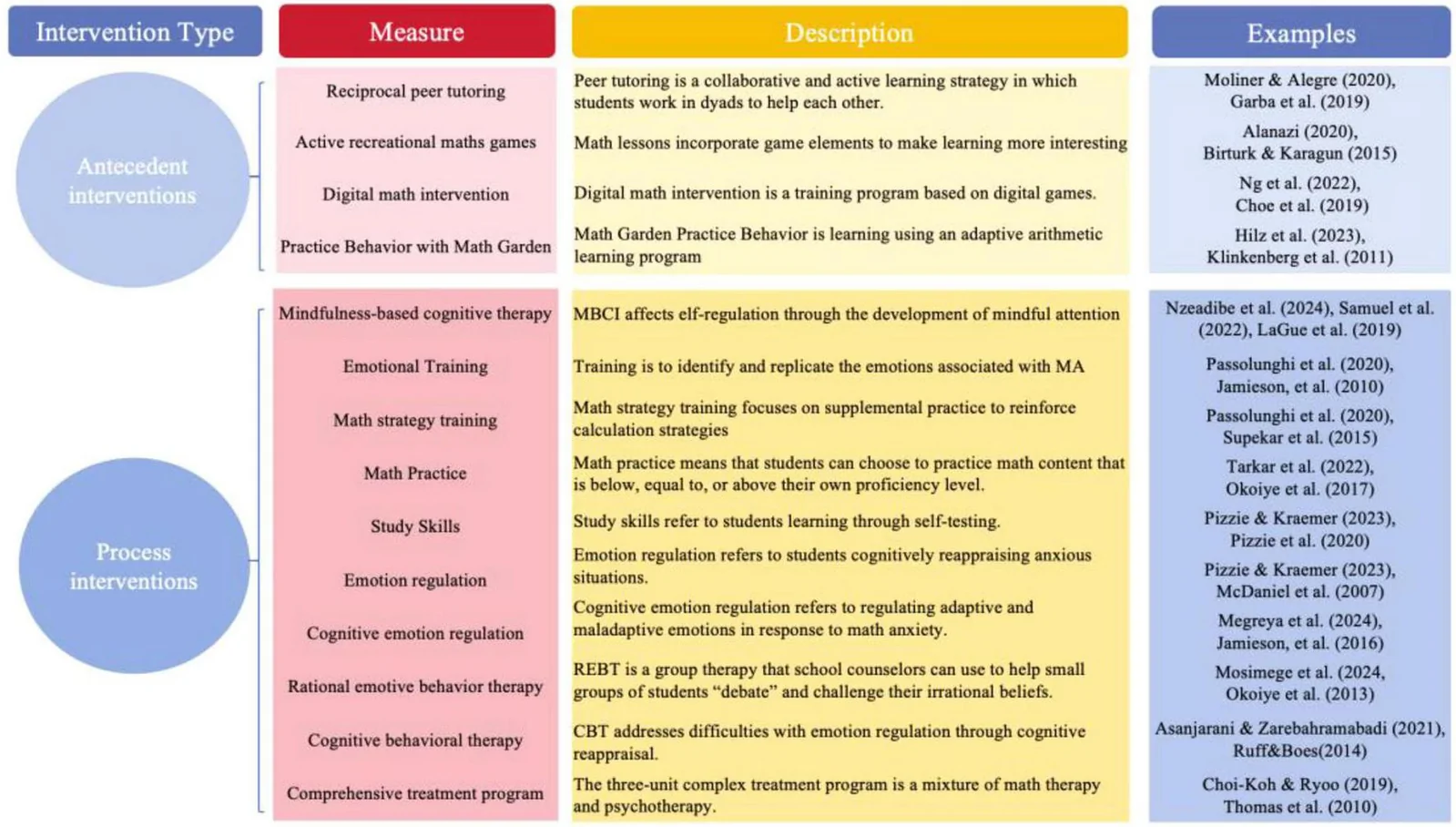
Interventions for alleviating mathematics anxiety.

The results of the strategy coding analysis of interventions for mathematics anxiety based on antecedents and processes of the ICILS framework are shown in Figure 4. Antecedent interventions mainly involved the learning environment, external support, and resources (e.g., peer tutoring, digital intervention, and gamification learning). Process interventions focused on cognitive, emotional, and behavioral adjustments (e.g., cognitive behavioral therapy, emotional regulation, and learning skills training). Antecedent interventions provided background conditions for the occurrence of mathematics anxiety, while process interventions directly acted on anxiety relief and improvement of learning outcomes.

## 5 Discussion

This scoping review examined mathematics anxiety research in K-12 education from January 2019 to December 2025, analyzing influencing factors, methodologies, and interventions to establish the current state of research and inform future research.

### 5.1 The factors affecting mathematics anxiety

#### 5.1.1 The antecedent factors

Grounded in the ICILS 2018 framework, this review identified a broad set of antecedent factors influencing mathematics anxiety in K–12 settings. At the individual level, gender and grade level were the most frequently examined predictors. Gender differences, often interpreted through stereotype threat, remained prominent (Wang, 2020; Milovanović, 2020; Xie et al., 2019), while age- and grade-related variation was widely documented (Luttenberger et al., 2018; Zhang et al., 2019). However, empirical attention has been heavily skewed toward elementary students. High school populations remain underrepresented, largely due to data collection challenges linked to intensive curricula and examination pressures (Barroso et al., 2021; Zhang et al., 2019). This gap constrains efforts to map developmental trajectories and to test whether antecedent effects vary across educational stages. Cross-grade research is further hindered by methodological complexities in sampling design, measurement invariance, and longitudinal tracking (Radević and Milovanović, 2023; Wu et al., 2012; Zhu et al., 2024), underscoring the need for rigorous, multi-cohort designs.

Environmental antecedents, particularly family and school contexts, were robust correlates of mathematics anxiety. Parental expectations, attitudes, and teachers’ instructional styles were consistently implicated (Li et al., 2021; Szczygieł, 2020; Zhou et al., 2020). Peer influences, though less directly examined, emerged as a plausible pathway. Peer comparison, competition, and cooperation shape classroom climate and students’ self-perception (Moliner and Alegre, 2020; Kim et al., 2023), yet it remains unclear whether peer dynamics operate as independent predictors or as moderators of other risk factors.

Culturally, the rapid growth of research in China highlights how academic pressure becomes institutionalized (Eidlin-Levy et al., 2023; Zhang et al., 2019). Nevertheless, global representation remains imbalanced, limiting generalizability and cross-national comparability (Radević and Milovanović, 2023; Ramirez et al., 2018). Large-scale, cross-cultural datasets with harmonized measures are needed to disentangle cultural norms from structural features of schooling.

#### 5.1.2 The process factors

Process factors represent the proximal mechanisms through which distal antecedents translate into mathematics anxiety. Behavioral factors, such as learning engagement and persistence, were shown to buffer anxiety by enhancing mastery experiences (Broda et al., 2023; Du et al., 2021). Conversely, disengagement and avoidance amplified negative affect. Cognitive factors critically shaped how students appraised and solved mathematical tasks. Metacognitive awareness enabled students to identify difficulties and deploy adaptive strategies, thereby reducing anxiety (LaGue et al., 2019; Desender and Sasanguie, 2022). Emotional factors, particularly mathematics attitude and interest, were closely tied to anxiety trajectories (Mammarella et al., 2023; Passolunghi et al., 2020). Negative attitudes predicted higher anxiety, whereas positive emotional engagement exerted protective effects.

Other process-related variables, such as arithmetic fluency and working memory, further modulated anxiety experiences (Codding et al., 2023; Van Mier et al., 2019). Importantly, these factors rarely operated in isolation. Behavioral, cognitive, and emotional processes interacted dynamically during learning, jointly shaping students’ mathematics-related emotional experiences (Escalera-Chávez et al., 2016). Future empirical work should move beyond variable-centered approaches to model these interactions using person-centered or multilevel designs.

#### 5.1.3 The outcome factors

Mathematics anxiety consistently predicted lower self-efficacy, reduced engagement, and poorer academic performance (Cribbs et al., 2021; Eidlin-Levy et al., 2023; Quintero et al., 2022). Moderating variables such as grade, gender, and prior achievement qualified these effects (Özcan and Eren Gümüş, 2019; Zhang et al., 2019). Recent studies also examined how mathematics anxiety interacts with other constructs, such as self-efficacy, teacher support, and working memory, to jointly influence achievement (Ching, 2017; Cribbs et al., 2021). A notable challenge in synthesizing the literature was the presence of contradictory findings regarding the strength and direction of associations between mathematics anxiety and academic outcomes. Although most studies report a negative correlation, several suggest negligible or context-dependent effects (e.g., Song et al., 2021; Caviola et al., 2022). These discrepancies likely stem from methodological variations, including differences in anxiety measurement scales, sample age ranges, and the extent of statistical control for cognitive covariates such as working memory.

Beyond immediate academic outcomes, mathematics anxiety shaped longer-term educational trajectories. Students with higher anxiety were more likely to avoid STEAM-related majors and careers (Ahmed, 2018; Eidlin-Levy et al., 2023). These findings indicate that outcome factors extend beyond classroom performance to influence identity formation and opportunity structures. Notably, negative outcomes may feed back into antecedent conditions, for example, by reinforcing low self-efficacy or narrowing course-taking patterns, potentially sustaining anxiety over time. Longitudinal research is required to test these recursive pathways.

### 5.2 The methodological characteristics of mathematics anxiety

#### 5.2.1 The analytical methods for mathematics anxiety

This review identified a predominant reliance on variable-centered analytical approaches, emphasizing associations between mathematics anxiety and macro-level outcomes such as academic performance and attitudes (Barroso et al., 2021; Hickendorff et al., 2018; Luttenberger et al., 2018). These approaches effectively capture general patterns but are less sensitive to individual heterogeneity. Although person-centered methods (e.g., latent profile analysis) remain underused, their emergence signals growing recognition of within-group variability (González-Gómez et al., 2023; Williams et al., 2024). Future empirical work should combine variable- and person-centered analyses to simultaneously model general trends and individualized pathways.

#### 5.2.2 The research methods for mathematics anxiety

Quantitative cross-sectional surveys dominated the literature, with only two mixed-methods studies identified. While cost-effective and scalable, this design limits causal inference and developmental understanding (Blok and Saris, 1983; Spector, 2019). Experimental studies, though rare, provided valuable insights into real-time cognitive mechanisms (Kreibig, 2010; Ramirez et al., 2018). The scarcity of longitudinal designs reflects practical challenges (e.g., attrition, cost) but constitutes a major gap, as it prevents researchers from modeling how anxiety evolves alongside learning processes. Similarly, the near absence of qualitative inquiry restricts insight into students’ lived experiences and classroom dynamics (Braun and Clarke, 2006; Hammarberg et al., 2016). A balanced portfolio that integrates longitudinal tracking, controlled experiments, and qualitative case studies is essential for capturing both the stability and fluidity of mathematics anxiety.

#### 5.2.3 The measurement tools for mathematics anxiety

Self-report questionnaires remained the primary measurement tool, with considerable diversity in scale focus (e.g., emotional experience vs. performance-related anxiety) (Barroso et al., 2021; Núñez Peña and Guilera Ferré, 2023). While practical and widely validated, self-report questionnaires are susceptible to recall bias and social desirability, and few primary studies incorporate objective cognitive assessments or physiological indicators. Recent studies have supplemented self-reports with physiological (heart rate, pupil dilation) and neuroimaging measures (EEG, fMRI), advancing understanding of anxiety’s embodied dimensions (Klados et al., 2019; Pizzie et al., 2020). However, these methods remain technically demanding and underused in K–12 samples. Future research should pursue multimodal measurement frameworks that integrate subjective reports, behavioral indicators, and physiological/neural data ICILS 2018 framework to more rigorously test hypothesized mechanisms.

### 5.3 The interventions for mathematics anxiety

Guided by the, interventions were categorized into antecedent-focused and process-focused approaches (Fraillon et al., 2018; Luttenberger et al., 2018). Antecedent-focused interventions sought to modify the contextual conditions under which mathematics learning occurs. For example, gamified classroom environments and math games informed by embodied cognition principles significantly reduced mathematics anxiety while improving performance (Alanazi, 2020; Hilz et al., 2023). Process-focused interventions targeted behavioral, cognitive, and emotional regulation strategies. Strategy training—such as algorithmic reasoning, emotion regulation, and self-testing—effectively alleviated anxiety by enhancing students’ sense of control and adaptive responding (Codding et al., 2023; Passolunghi et al., 2020).

While both approaches demonstrated empirical support, they were typically implemented in isolation. Antecedent-focused interventions reduced exposure to anxiety-provoking conditions, whereas process-focused interventions strengthened individual coping capacities. This separation limits understanding of how environmental supports and personal strategies interact. Moreover, most intervention studies were short-term, small-scale, and lacked long-term follow-up. Future research should prioritize multimodal interventions that integrate contextual modification and strategy instruction, employ rigorous designs (e.g., randomized controlled trials, longitudinal tracking), and evaluate both immediate anxiety reduction and downstream academic outcomes.

## 6 Limitations

Several limitations should be noted. First, as a scoping review, this study mapped the breadth of K–12 mathematics anxiety research without appraising methodological quality or estimating effect sizes; thus, no claims regarding intervention efficacy or association magnitude can be made.

Second, restricting searches to article titles enhanced specificity but may have excluded relevant studies mentioning mathematics anxiety only in abstracts, keywords, or full texts. This limitation, compounded by the file drawer problem, may have biased the synthesis toward statistically significant findings.

Third, limiting inclusion to English-language, peer-reviewed empirical articles may have omitted studies published in other languages or in gray literature. Given the cultural embeddedness of mathematics anxiety, this constraint may limit the generalizability of the proposed framework across diverse educational systems.

Finally, included studies were predominantly from China and Western contexts, with scarce representation from Africa, Latin America, and Southeast Asia. This geographic skew likely reflects global disparities in research infrastructure and publication practices rather than true regional differences in mathematics anxiety. Such imbalance may limit the cross-cultural generalizability of our framework. Future reviews should adopt culturally inclusive search strategies to address this gap.

## 7 Conclusion and implications

This scoping review synthesized 132 studies on mathematics anxiety in K–12 education (2019–2025). Grounded in the ICILS 2018 framework, it mapped the literature across antecedent, process, and outcome domains. Individual and environmental antecedents were predominant, yet research on high school students and cross-grade comparisons remained limited. Process research emphasized behavioral, cognitive, and emotional mechanisms, while outcome studies consistently linked anxiety to reduced performance and constrained educational trajectories. Methodologically, the field remained reliant on variable-centered, cross-sectional designs, with intervention research split between contextual restructuring and strategy training. Moving forward, research should transition from isolated predictors to integrated, systems-based models that explicitly link antecedents, learning processes, and long-term outcomes. This requires diversifying methodologies through person-centered analyses, longitudinal tracking, and multimodal measurement (e.g., eye tracking, EEG, fMRI). To translate these findings into practice, educators and policymakers should implement dual-focus interventions that pair anxiety-reducing classroom environments with explicit self-regulation training. This imperative is particularly urgent for high school students navigating critical educational transitions.

## Data Availability

The original contributions presented in this study are included in the article/Supplementary material, further inquiries can be directed to the corresponding author.
