# Supplementary material for: What shapes mathematics anxiety in K-12 education? A scoping review of research from 2019 to 2025
Source: Front Psychol. 2026 Aug 6;17:1894090. doi: 10.3389/fpsyg.2026.1894090 (PMC13490170; doi:10.3389/fpsyg.2026.1894090)
Supplement: Supplementary file 1 [file Table_1.docx]

**Table**

| **Variable** | **code** | **Description** |
| --- | --- | --- |
| Title |  | The article’s title |
| Authors |  | Author’s names |
| Publication year | 2019/2020/2021/2022/2023/2024 |  |
| Country | USA/UK/China,…. | country of participants |
| Research methods | quantitative/qualitative/mixed | case study, investigate,  (quasi-)experimental,observe |
| Participants’s level of education | Elementary/Middle /High | elementary school students  middle school students  high school students |
| Sample size | 0-50/51-100/100-200/201-500/501-1,000/>1,000 |  |
| Level of math anxiety | No classification/ Individual-centered classification/variable-centered classification | no classification means math anxiety as a total. individual-centered classification means math anxiety with low, median, high concerning the individual. variable-centered classification means math anxiety with low, median, high concerning the variable |
| Design | cross-sectional studies/longitudinal studies | cross-sectional studies considered a single time  longitudinal studies considered a long-time |
| The scale of math anxiety | AMAS(Hopko(2003))/AMAS(Richardson & Suinn (1972))/ PISA 2012…… | the study measures math anxiety in different scale |
| Antecedents | antecedents are exogenous factors that condition the ways in which mathematics anxiety takes place. They are contextual factors that are not directly influenced by learning-process variables or outcomes. | gender/grade/environment factor(parents,teachers,peers) |
| Processes | processes are those factors based on behavior, emotion, cognition and others that directly influence mathematics anxiety | critical thinking  /mathematics attitude/  mathematics self-effective….. |
| Outcomes | outcomes are those factors that they are directly influenced by mathematics anxiety | mathematics acheievement/math motivation/career choice... |
| Intervention | interventions are those strategies based antecedent and process that can reduce mathematics anxiety | peer tutoring/  arithmetic strategies... |
